# Supplementary material for: Learning curve and surgical outcome of robotic assisted colorectal surgery with ERAS program
Source: Sci Rep. 2022 Nov 29;12:20566. doi: 10.1038/s41598-022-24665-w (PMC9709162; doi:10.1038/s41598-022-24665-w)
Supplement: Supplementary file 2 — Supplementary Information 2. [file 41598_2022_24665_MOESM2_ESM.zip › RAL Raw data-Ñ[▒K/070/Patho_ñw╝╨░O▒Kñσ.pdf]

臺中榮民總醫院 臺中市臺灣大道四段1650號 病理部活體組織切片 臨床醫學實驗及病理診斷中心 檢驗(查)報告

[redacted]

Pathologic diagnosis:
1. Rectum, robotic assisted LAR --- a. Status post endoscopic mucosal resection with submucosal fibrosis and congestion. No residual invasive carcinoma found. The bilateral cut ends and perirectal lymph nodes (0/18) are free. b. Tubular adenoma.
2. IMA root, dissection --- Unremarkable fibrovascular tissue.
3. Rectum, cut end, distal, resection --- Chronic inflammation.

Prognostic and predictive factor:
1. Degree of dysplasia: Low grade.
2. Margins: Free.

Gross description:
The specimen consists of 1) a segment of colon, 10 cm in length and 4.5 cm in proximal cut end circumference, 6 cm in distal circumference, and perirectal soft tissue. There is area of erythromatous change labeled with a clip, 1.5 cm to the nearest cut end. On cross section, only discoloration of the submucosa is seen, but no invasive mass lesion can be identified grossly. There is another polypoid lesion, 0.5x0.3 cm, noted over the mucosa. The both cut ends are free of tumor grossly. 2) IMA root, a piece of tan yellow soft tissue, 3x2.1x0.8 cm. 3) distal cut end, a piece of tan mucosa-covered soft tissue, 2.6x1.6x0.8 cm. Representative part for section: A) proximal cut end B) distal cut end C-E,U-Y) clip labeled mucosa (inked green) F) polyp G-H) specimen 2-3 J-T) lymph nodes.
#T-59600_2 #M-82110_2 0 1105 000000
#T-C4560_2 #M-09183_2 0 1105 000000

已電子簽章

敬告：本單僅供醫師參考使用，不具任何申請效力！！
請病患妥為保管，若不慎遺失需自負個人資料外洩責任！
